# A Natural Experiment on the Condition-Dependence of Achromatic Plumage Reflectance in Black-Capped Chickadees

**DOI:** 10.1371/journal.pone.0025877

**Published:** 2011-10-03

**Authors:** Liliana D'Alba, Caroline Van Hemert, Colleen M. Handel, Matthew D. Shawkey

**Affiliations:** 1 Department of Biology and Integrated Bioscience Program, University of Akron, Akron, Ohio, United States of America; 2 U. S. Geological Survey, Alaska Science Center, Anchorage, Alaska, United States of America; 3 Department of Biology and Wildlife, University of Alaska Fairbanks, Fairbanks, Alaska, United States of America; Arizona State University, United States of America

## Abstract

Honest advertisement models posit that only individuals in good health can produce and/or maintain ornamental traits. Even though disease has profound effects on condition, few studies have experimentally tested its effects on trait expression and even fewer have identified a mechanistic basis for these effects. Recent evidence suggests that black and white, but not grey, plumage colors of black-capped chickadees (*Poecile atricapillus*) are sexually selected. We therefore hypothesized that birds afflicted with avian keratin disorder, a condition that affects the beak and other keratinized tissues, would show reduced expression of black and white, but not grey, color. UV-vis spectrometry of black-capped chickadees affected and unaffected by avian keratin disorder revealed spectral differences between them consistent with this hypothesis. To elucidate the mechanistic bases of these differences, we used scanning electron microscopy (SEM), electron-dispersive x-ray spectroscopy (EDX) and a feather cleaning experiment. SEM showed extreme feather soiling in affected birds, and EDX revealed that this was most likely from external sources. Experimentally cleaning the feathers increased color expression of ornamental feathers of affected, but not unaffected, birds. These data provide strong evidence that black and white color is an honest indicator in chickadees, and that variation in feather dirtiness, likely due to differences in preening behavior is a mechanism for this association.

## Introduction

Honest advertisement models posit that expression of ornamental traits should be linked to the overall quality of an organism [1]–[3]. If expression of a trait is condition-dependent, then high-quality individuals should suffer a lower fitness cost than low-quality individuals for the same expression of the trait [3], [4]. Numerous studies have demonstrated associations between plumage color and various aspects of quality, including nutritional condition [5]–[9], parasite resistance [10], territory quality [11], [12], parental effort [13], and social status (reviewed in [14]). However, only a few studies [15]–[18] have examined the effects of disease on plumage color. Such research is important because disease state can serve as a reliable metric of overall physiological condition. Furthermore, when the physiological effects of the disease are known, it may be possible to establish a mechanistic link between expression of color and disease state, increasing certainty of cause-and-effect relationships [16].

**Figure 1:**
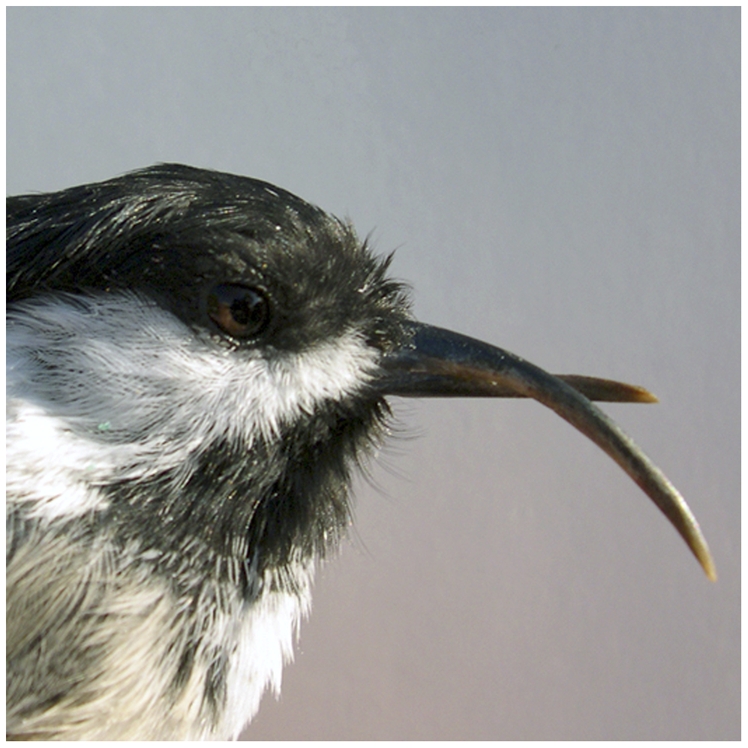
Black-capped chickadee affected by avian keratin disorder. An individual from an Alaskan population of black-capped chickadees shows Avian keratin disorder. The disease produces elongated and crossed beak phenotypes.

Outbreaks of disease thus provide natural experiments that can be exploited to address the hypotheses of honest advertisement models. An epizootic termed avian keratin disorder has recently been documented among black-capped chickadees (*Poecile atricapillus*), northwestern crows (*Corvus caurinus*), and other avian species in Alaska and the Pacific Northwest region of North America [19], [20]. This condition results in deformation of the beak (figure 1), and may be accompanied by lesions in other keratinized tissues of the skin, legs, feet, claws, and feathers [19], [20]. Affected birds have difficulty feeding and preening, have dirty and matted plumage, and suffer elevated incidence of parasitic feather mites [19], all of which likely have detrimental effects on individual health and fitness.

Recent evidence suggests that the contrasting black and white color patches of black-capped chickadees are sexually selected. Males with darker and more UV-reflective black plumage are more dominant [21], [22] and those with brighter white plumage and more UV-reflective black plumage have higher reproductive success [23]. By contrast, grey feathers are not associated with either parameter [21], suggesting that they are not sexually selected.

A population of black-capped chickadees affected by avian keratin disorder provides an opportunity to directly assess the reliability of achromatic plumage as a health indicator. Here we use ultraviolet-visible spectrometry to examine achromatic plumage reflectance in this population, predicting that affected birds would have reduced black and white color, but similar grey color, to unaffected birds. The difficulties that affected birds have in preening [19] led us to further hypothesize that this difference would be caused by increased dirtiness of affected birds' feathers. We tested this hypothesis using scanning electron microscopy (SEM), energy-dispersive x-ray spectroscopy (EDX) and a washing experiment. Dirt can dramatically alter reflectance, particularly in the UV spectrum [24], so this could represent a mechanistic link between the expression of plumage reflectance and individual condition in chickadees.

## Results

### (a) Feather appearance

**Figure 2:**
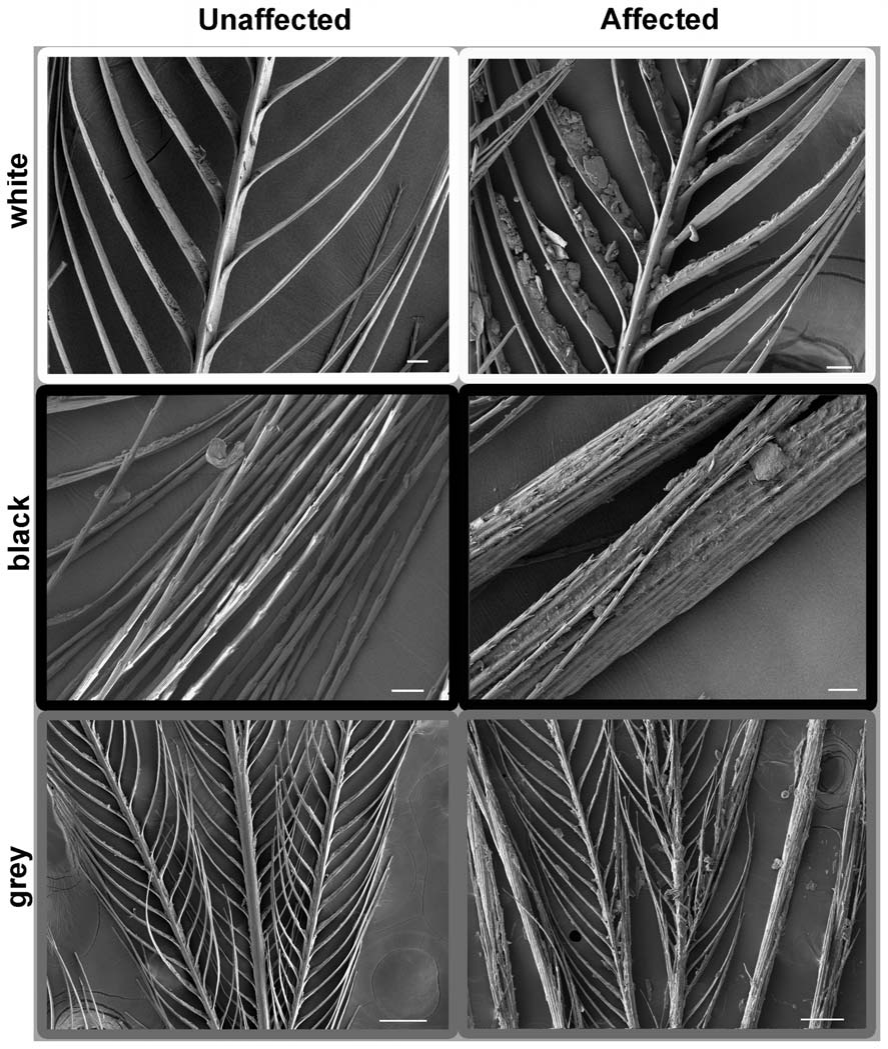
Feather microstructure of affected and unaffected birds. Examples of SEM micrographs of white cheek feathers (upper panels) black bib feathers (middle panels) and grey mantle feathers (lower panels) from black-capped chickadees unaffected (left) and affected (right) by avian keratin disorder. Scale bars are 20 µm.

**Figure 3:**
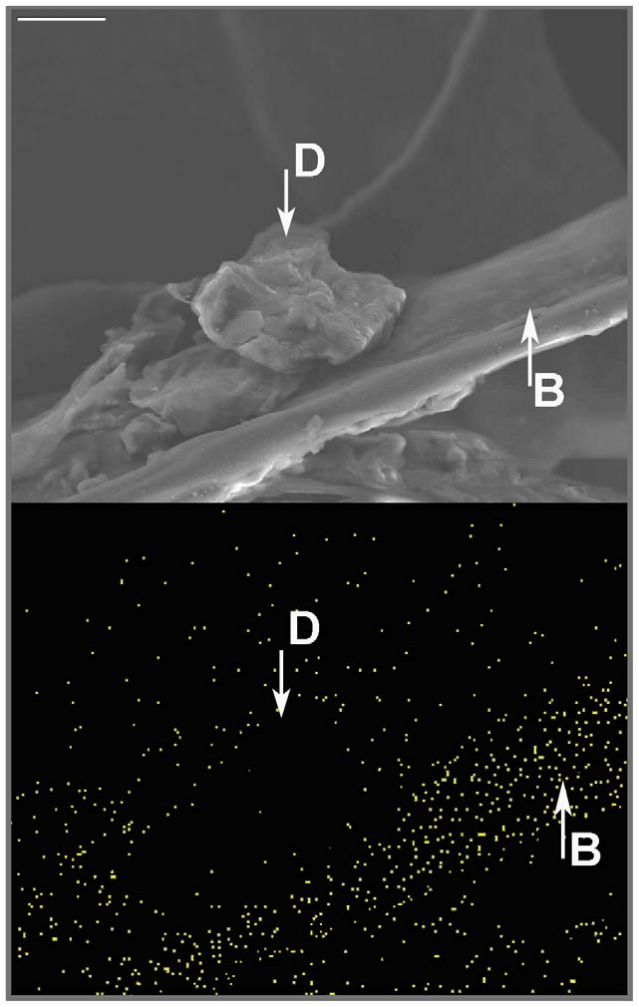
Soiling on black-capped chickadee feathers. Micrograph of soiled barb of a white black-capped chickadee feather (upper panel) and corresponding EDX dot map of sulfur (lower panel). Yellow dots in bottom panel indicate the presence of sulfur corresponding to keretinous structures. *d* = debris, *b* = barb. Scale bar is 10 µm.

**Figure 4:**
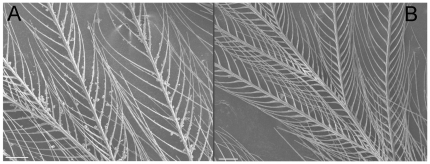
Removal of feather soling by the ethanol treatment. Example of a white feather from a black-capped chickadee affected by avian keratin disorder before (*a*) and after (*b*) washing with 50% ethanol. Scale bar is 100 µm.

Feathers of affected birds had a matted appearance that was evident to the naked eye. SEM revealed clear differences in appearance of feathers from affected and unaffected birds. White, black, and grey feathers of affected birds all had large deposits of debris on barbs and barbules that strongly contrasted with the clean feathers of unaffected birds (figure 2). In some cases, barbules in black feathers were so heavily soiled that they were stuck together. EDX analysis detected sulphur in adjacent barbs and barbules but none in the debris (figure 3), confirming that the debris was not keratinous in nature. SEM examination demonstrated that our ethanol washing treatment successfully removed the debris from feathers (figure 4).

### (b) Color measurements before the washing treatment

**Figure 5:**
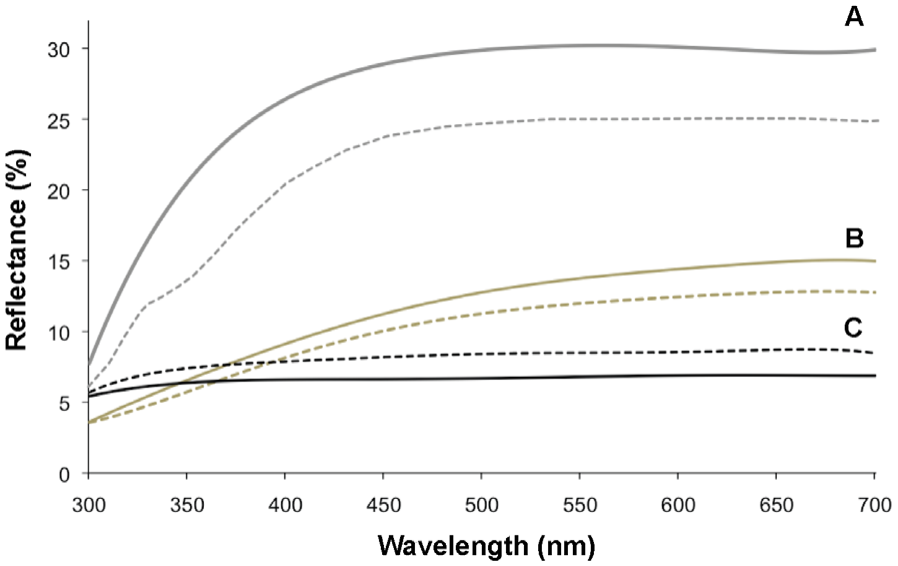
Spectral curves of feathers of affected and unaffected black-capped chickadees. Mean plumage reflectance curves for white A), grey B), and black C) body regions of birds unaffected (solid lines; *n* = 10) and affected (dashed lines; *n* = 10) by avian keratin disorder.

Reflectance curves for white, black and grey feathers were similar but slightly lower than those measured previously in the same species [23], [21] (figure 5), likely because our measurements were performed on taped feathers on a black background rather than directly on the bird (e.g. [21]).

#### White feathers

**Figure 6:**
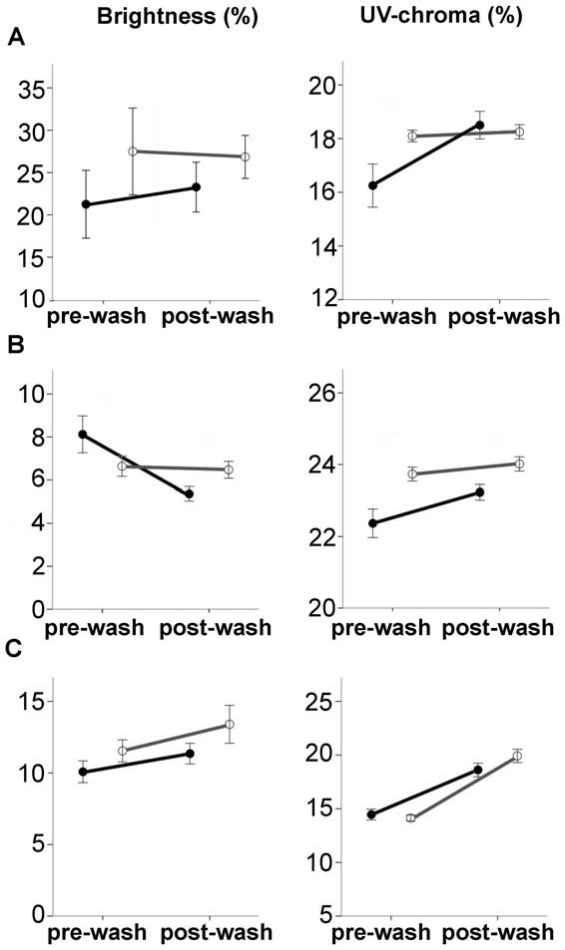
Effects of the ethanol wash on spectral characteristics of feathers. Brightness and UV-chroma before and after the ethanol wash treatment of (*a*) white, (*b*) black and (*c*) grey feathers of black-capped chickadees affected (filled symbols) and unaffected (open symbols) by avian keratin disorder. Values are presented as mean ± SE.

Brightness of white feathers was slightly lower for affected than unaffected birds but did not differ significantly (table 1; figure 6*a*). Effect size of disease state on UV-chroma of white feathers was large (>0.8): UV-chroma was significantly lower in affected than unaffected birds, and did not differ by sex (table 1).

**Table 1:** GLM showing effects of disease state (affected versus unaffected by avian keratin disorder) and sex on color variables of white, black, and grey feathers of black-capped chickadees before wash treatment.

	Brightness				UV chroma			
	*F*	*df*	*p*	*d* (95%CI)	*F*	*df*	*p*	*d* (95%CI)
***White***								
Disease state	3.60	1,18	0.07	0.76 (0.71, 0.81)	**4.9**	**1,18**	**0.04**	**1.04 (1.04, 1.06)**
Sex	1.49	1,17	0.29	0.29 (0.27,0.35)	0.04	1,17	0.84	0.1 (0.09, 0.12)
***Black***								
Disease state	**5.10**	**1,18**	**0.03**	**1.07 (1.05, 1.07)**	**9.40**	**1,18**	**0.007**	**1.45 (1.45, 1.46)**
Sex	1.48	1,17	0.24	0.28 (0.26, 0.29)	1.15	1,17	0.29	0.17 (0.16, 0.17)
***Grey***								
Disease state	1.80	1,18	0.19	0.58 (0.57, 0.6)	0.31	1,18	0.58	0.26 (0.25, 0.27)
Sex	0.05	1,17	0.82	0.02 (0, 0.03)	1.73	1,17	0.20	0.13 (0.13, 0.14)

Measure of effect size for main terms in the model is Cohen's *d*. Non-significant terms were stepwise-removed from the model. Numbers in bold denote significance at the 5% level.

#### Black feathers

Brightness of black bib feathers was higher in affected than unaffected birds (table 1; fig. 6b), and there was no difference by sex (table 1). UV-chroma of black feathers was lower in affected than unaffected birds (fig. 6b) and did not differ by sex (table 1). The effect size of disease state of both brightness and UV-chroma was large (>0.8) compared to the effect size of sex (<0.3; table 1).

#### Black and white contrast

**Figure 7:**
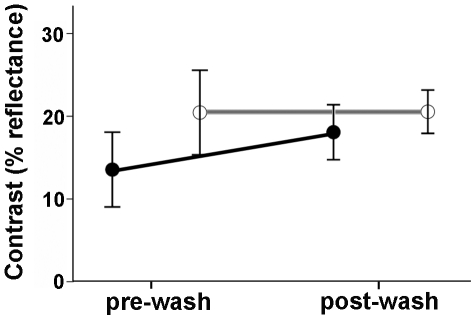
Effects of the ethanol wash on contrast in brightness between black and white feathers. Means ± SE of contrast between black and white feathers of black-capped chickadees affected (filled symbols) and unaffected (open symbols) by the keratin disorder before and after the ethanol wash treatment.

Contrast in brightness between black and white feathers was significantly lower for affected (mean = 13.0±2.4%, *n* = 10) than unaffected (mean = 20.8±2.4%, *n* = 10) birds before washing (*F*_1,18_ = 4.09, *p* = 0.05, figure 7). Sex differences were not significant (*F*_1,17_ = 1.34, *p* = 0.18). The effect size of disease state on contrast was high (*d* = 0.95, 95%CI = −4.03 to 5.81) compared to the effect size of sex (*d* = 0.40, 95%CI = −5.81 to 4.1).

#### Grey feathers

Neither brightness nor UV-chroma of grey feathers differed by disease state or sex (table 1; figure 6c). The effect size of disease state and sex on both spectral variables was moderate (<0.5) to small (<0.2) (table 1).

### (c) Wash experiment

#### White feathers

Brightness of white feathers of affected birds did not change (*t*_9_ = 0.79, *p* = 0.44) but their UV-chroma increased by 2.3±0.7% (*t*_9_ = 3.29, *p* = 0.009; figure 6a) after washing to reflectance levels statistically indistinguishable from those of unaffected birds. Neither brightness nor UV chroma of unaffected birds was changed by the treatment (brightness *t_9_* = 0.05, *p* = 0.96; UV-chroma *t_9_* = 0.42, *p* = 0.68; figure 6a). After washing, there were no significant differences in brightness or UV-chroma of white feathers by disease state or sex (table 2); however, the effect size of disease state on brightness was large (>0.8; table 2).

**Table 2:** GLM showing effects of disease state (affected versus unaffected by avian keratin disorder) and sex on color variables of white, black and grey feathers of black-capped chickadees after removal of dirt through wash treatment.

	Brightness				UV chroma			
	*F*	*df*	*p*	*d* (95%CI)	*F*	*df*	*p*	*d* (95%CI)
***White***								
Disease state	3.36	1,18	0.08	0.86 (0.84, 0.89)	0.18	1,18	0.67	0.2 (0.19, 0.21)
Sex	0.76	1,17	0.39	0.22 (0.18, 0.24)	0.08	1,17	0.78	0.09 (0.09, 0.1)
***Black***								
Disease state	**4.58**	**1,18**	**0.04**	**1.01 (1, 1.02)**	**7.16**	**1,18**	**0.01**	**1.26, (1.26, 1.27)**
Sex	0.003	1,17	0.95	0.16 (0.15, 0.16)	0.09	1,17	0.76	0.09 (0.09, 0.1)
***Grey***								
Disease state	1.84	1,18	0.19	0.57 (0.55, 0.59)	2.18	1,18	0.15	0.38 (0.36, 0.39)
Sex	0.67	1,17	0.42	0.24 (0.23, 0.27)	0.81	1,17	0.37	0.27 (0.26, 0.28)

Measure of effect size for main terms in the model is Cohen's *d*. Non-significant terms were stepwise-removed from the model. Numbers in bold denote significance at the 5% level.

#### Black feathers

Black feathers of affected birds decreased in brightness (mean difference −2.75±0.97%, *t*_9_ = 2.81, *p* = 0.02; figure 6b) and increased in UV-chroma (mean difference 0.86±0.35%, *t*_9_ = 2.44, *p* = 0.03; figure 6b) after the wash. By contrast, black feathers of unaffected birds did not change in either color parameter (brightness *t*_9_ = 0.30, *p* = 0.76, UV-chroma *t*_9_ = 1.39, *p* = 0.19; figure 6b). After cleaning, black feathers of unaffected birds had both higher brightness and UV-chroma than those of affected birds, and there was no effect of sex. Effect sizes of disease state on both spectral variables was very large (>1.0; table 2), while the effect size of sex was small (<0.2; table 2).

#### Black and white contrast

Contrast in brightness between black and white feathers showed little change with the washing treatment (affected: *t*_9_ = −2.03, *p* = 0.07; unaffected: *t*_9_ = 0.006, *p* = 0.99; figure 7) and after the wash, did not differ by disease state or sex (*F*_1,18_ = 1.36, *p* = 0.25; sex *F*_1,17_ = 0.62, *p* = 0.44). The effect sizes of disease state and sex on contrast were moderate and small respectively (disease state *d* = 0.95, 95%CI = −4.03 to 5.81; sex *d* = 0.25, 95%CI = −3.3 to 2.59).

#### Grey feathers

Brightness of grey feathers did not change in response to the washing treatment (affected: *t*_9_ = −1.08, *p* = 0.30, unaffected: *t*_9_ = −1.33, *p* = 0.21; figure 6c) and still did not differ between affected and unaffected birds (table 2). However, UV-chroma increased by 4.1±0.82% and 5.7±0.68% in feathers of affected (*t* = −5.02, *p* = 0.001) and unaffected (*t*_9_ = −8.41, *p*<0.001) birds, respectively; the difference between the groups was not significant and the effect size of disease state and sex on both spectral variables was moderate (<0.5) to small (<0.2) (table 2, figure 6c).

## Discussion

Our results strongly support the hypothesis that ornamental black and white plumage color reflects disease state in black-capped chickadees and thus honestly reveals information about an individual's disease state. As predicted, color of black and white feathers differed between affected and unaffected birds, but color of control non-ornamental grey feathers did not. Our data further suggest that feather soiling, likely a result of reduced preening ability due to overgrowth of the beak [19], is a proximate cause of these color differences. This is one of the few studies to not only demonstrate a link between disease state and color but also to provide a mechanistic explanation for that link.

Natural experiments like this are uniquely powerful in that they allow us to directly observe how natural processes occur in a real-world context. While they do not afford the same level of control as traditional experiments, the obvious and strong effects of the “treatment” (disease) and the mechanistic connection between it and the color change we observed (as determined by an additional lab-based experiment) make it reasonable to infer that they are connected.

UV-reflectance is a signal used in mate choice (e.g., starlings *Sturnus vulgaris*, [25]; bluethroats *Luscinia svecica svecica*, [26]; pied flycatchers *Ficedula hypoleuca*, [27]; budgerigars *Melopsittacus undulatus*, [28]), but the mechanisms linking color to individual condition are still unclear [29], [30], [31]. Here, we found that only healthy birds maintain clean plumage and that cleanliness affects UV reflectance of the ornamental trait, suggesting that it honestly reflects health. In contrast with Mennill et al. [21], we did not detect sexual dichromatism in plumage brightness, which might be partly explained by our small sample size relative to Mennill et al. [21]) and/or in differences in the methods used to calculate reflectance (average percent reflectance here as opposed to Principal Component Analysis of average reflectance curves). Nevertheless, the strong effect of disease state (compared to the effect of sex) on plumage brightess observed here suggests that clean and bright plumage may be used by both males and females to assess a potential mate's condition.

Feather colors can change between the time of feather growth and the time that they are advertised as a result of UV damage, abrasion or breakdown by abiotic and biotic factors [32]–[37]. The integrity of plumage color can be energetically costly to maintain and should thus be a reliable communication signal. For example, experimental breakdown of feathers by bacteria decreases UV-chroma in structurally colored blue feathers [36], suggesting that their bright UV colors honestly signal abundance of feather-degrading bacteria to potential mates. Similarly, UV-chroma of black feathers appears to provide a robust signal of disease state in black-capped chickadees.

Our SEM data and washing experiment demonstrated that a significant portion of the observed color differences between unaffected and affected birds was caused by soiling. Effects of soiling on plumage color have been investigated in a few studies but results vary with the composition of the debris, plumage coloration, and species. For example, soiled carotenoid-colored feathers were less bright [38], [39] while soiled white breast feathers [37] were more UV-chromatic, than cleaned feathers. Conversely, experimental soiling and natural buildup of dirt and waxes decreased UV-reflectance, of structurally-colored blue feathers [30], [40] and iridescent feathers [41]. These latter results and ours are consistent with the idea that dirt differentially absorbs UV wavelengths [24] leading to lower UV-reflectance in soiled feathers. By contrast, increased brightness of soiled black feathers may result from incoherent light scattering by randomly aggregated particles reflecting at all wavelengths [42]. Alternatively, the debris that accumulates on black feathers may simply be closer to white and thereby reflect more light. This would further explain why dirt did not affect overall brightness of white feathers. Interestingly, soiling had no effect on grey feathers, suggesting that this color effectively conceals dirt and is thus not useful as an honest ornament.

Even after cleaning, spectral characteristics of black feathers remained distinct between affected and unaffected birds. This could be the result of an incomplete cleaning treatment or could suggest underlying morphological differences. For example, secondary effects of disease during feather growth could result in reduced volume or altered composition of melanin [43], [44] or microstructural components that influence feather brightness [45]. Potential differences in feather microstructure in relation to disease state warrant further investigation.

Of course, avian keratin disorder produces abnormal beak phenotypes that mark a bird as unhealthy even in the absence of plumage color differences. However, preening is an energetically costly activity [46], [47] that is rarely performed by diseased or otherwise unhealthy birds [48], [19]. Therefore, the effects we observe here may be generalizable to other systems, particularly those in which achromatic plumage has been associated with parameters of individual quality (e.g., pied flycatchers; [49]). However, comparisons of plumage expression across other species and disease states are needed to confirm this hypothesis. Future studies should consider the effect of plumage maintenance when investigating condition-dependent signals of color displays.

## Materials and Methods

### Ethics statement

The University of Alaska Fairbanks and the USGS Alaska Science Center institutional review boards (Animal Care and Use committees) approved this study (assurances nos. 07-49, 08-57) and we followed all applicable institutional guidelines.

### (a) Sample collection

In March–April 2009 we collected three contour feathers from three different color patches (black bib, white cheek, grey mantle) of 10 affected and 10 unaffected black-capped chickadees held captive at the University of Alaska Fairbanks for approximately five months as part of a separate study of avian keratin disorder. These birds were captured as adults from south-central and interior Alaska after fall moult; therefore, feathers used in this study were grown prior to captivity. All affected birds exhibited beak deformities and were classified according to the criteria established by [19]. Birds were captured using funnel traps and mist nets as described by [19]. We performed DNA analysis to determine sex of birds from blood samples drawn from the brachial vein [19], [50].

### (b) Feather appearance

We compared feather appearance of affected (*n* = 10) and unaffected (*n* = 10) captive birds using SEM. Single feathers were mounted on stubs with carbon tape, sputter-coated with silver and viewed on a scanning electron microscope (JSM7401F, JEOL Japan). Following these observations, we analyzed the composition of three unwashed feathers of affected birds using EDX to determine if the material observed on barbs and barbules was the product of abnormal accumulation of amorphous keratin produced by feather cells. This standard method uses x-rays emitted from the sample during bombardment by an electron beam to characterize the elemental composition of materials. Beta-keratins, which make up feathers, contain small amounts of sulphur that are detectable in this manner [51], [52].

### (c) Color measurements

We taped three feathers per color patch per individual to gloss-free black construction paper, and recorded spectral data from the distal portion of feathers using an AvaSpec 2048 spectrometer (range 250–880 nm, Avantes, Broomfield, CO, USA). We collected color data at normal (0° incident light/0° measurement) incidence using a bifurcated micron fiber optic probe held by a probe holder (RPH-1, Avantes) with matte black interior that excluded ambient light. All data were generated relative to a white standard (WS-2, Avantes). We used AvaSoft software (Avantes) to record and average 20 spectra sequentially, and recorded and averaged three measurements from randomly chosen points on each color sample. We calculated brightness as the average percent reflectance in the 300–700 nm range and UV-chroma as the proportion of total reflectance occurring within 300–400 nm. We used brightness and UV-chroma of white and black feathers because previous studies had shown that individual variation in these parameters is correlated with dominance status and reproductive success [21], [23]. Contrast in brightness within an individual bird's plumage (e.g., between adjacent white cheek and black bib patches) may enhance conspicuousness of signals [53] or, as shown for black-capped chickadees [21], constitute a signal itself, conveying individual information on sex and rank. We therefore included contrast of white cheek and black bib feathers as an additional component of plumage color. We calculated contrast as the difference between mean brightness values (% reflectance) of the two body regions.

We used spectral variables rather than those generated by avian color vision models both for ease of comparison with previous studies and because differences in feather appearance between affected and unaffected birds were evident with unaided human vision and therefore should be easily detectable by birds.

### (d) Washing experiment

To determine if color differences between affected and unaffected birds were caused by differences in soiling, we immersed black and white feathers from each individual in a solution of 50% ethanol for 5 min, rinsed them once with distilled water and allowed them to air dry in clean petri dishes for 2–3 hours. We examined a subsample of feathers with the SEM to confirm that this treatment was effective at removing debris. We then measured color of cleaned feathers using the methods described above.

### (e) Statistical analyses

Our data did not depart from normality; thus, we used parametric tests in all cases. To compare plumage reflectance between unaffected and affected birds we used general linear models (GLMs) with either brightness or UV-chroma as response variables and disease state (affected or unaffected by avian keratin disorder) and sex as explanatory variables. Because of limited statistical power, we did not test the interaction term of disease state and sex. We analyzed one model for each feather color (white, black or grey) and one model for contrast between black and white feathers. We report the main effects after non-significant (p>0.05) factors were removed. Effect size, which is less sensitive to sample size effects, was calculated for both main terms (disease state and sex) in the GLMs using Cohen's d [54]. To test the effect of washing on color variables we used paired *t*-tests in which brightness, UV-chroma and contrast measurements before and after the wash treatment for each individual were compared. All probabilities are two-tailed and values are reported as means ± SE. Analyses were performed in R, version 2.9.0 (R Development Core Team, Vienna).
